# Occurrence and Treatment Outcome of Late Complications After Free Fibula Flap Reconstruction for Mandibular Osteoradionecrosis

**DOI:** 10.7759/cureus.13833

**Published:** 2021-03-11

**Authors:** Junya Yamashita, Masaya Akashi, Daisuke Takeda, Junya Kusumoto, Takumi Hasegawa, Kazunobu Hashikawa

**Affiliations:** 1 Oral and Maxillofacial Surgery, Kobe University Hospital, Kobe, JPN; 2 Oral and Maxillofacial Surgery, Kobe University Graduate School of Medicine, Kobe, JPN; 3 Plastic Surgery, Kobe University Hospital, Kobe, JPN

**Keywords:** osteoradionecrosis of the jaw, free fibula flap, late complications, antibiotic

## Abstract

Purpose: This study aimed to evaluate the occurrence and treatment outcome of late complications after free fibula osteocutaneous flap reconstruction for mandibular osteoradionecrosis (ORN).

Methods: We enrolled 15 consecutive patients (14 men, one woman; median age 65 years, range 57-80 years) who underwent free fibula reconstruction for advanced mandibular ORN during 2013-2017 with two or more years of follow-up. Late complications included infection, plate exposure, and recurrence at the resection margin. The effect of perioperative antibiotic administration on late complications was also assessed.

Results: Late complications occurred in 33.3% (5/15) of patients, including two infections (local and distant), two plate exposures, and two recurrences (plate exposure and recurrence occurred in one patient). Perioperative antibiotic administration duration did not significantly affect the occurrence of postoperative late complications. All late complications were treated without problems.

Conclusions: Late complications after ORN reconstructive surgery are not uncommon, but can be treated properly.

## Introduction

Advances in radiation therapy (RT) have improved treatment outcomes in patients with head and neck malignancies (e.g., human papilloma virus-positive oropharyngeal squamous cell carcinoma [1]). This longer patient survival has made it more likely for late effects of RT to become evident [2]. These late adverse effects present serious oral complications, the most problematic of which is osteoradionecrosis (ORN) of the jaw. When the ORN worsens over time, it may manifest as pathological fractures, orocutaneous fistulae, persistent purulent drainage, and/or severe pain [3]. To control infection and avoid decreasing the patient’s quality of life, surgical interventions such as adequate debridement and simultaneous reconstruction with a composite free flap are essential [4].

Although there have been previous large-scale studies on the outcomes of free flap reconstruction for ORN of the jaw [5-7], long-term follow-up studies after such surgery are scarce. This study aimed to evaluate the occurrence and treatment outcome of late complications after free fibula reconstruction for mandibular ORN during routine postoperative observation of at least two years.

## Materials and methods

This retrospective study included 15 consecutive patients (14 men, one woman; median age 65 years, range 57-80 years) who underwent resection of advanced mandibular ORN with simultaneous free fibula flap transfer at Kobe University Hospital between July 2013 and May 2017. All flap harvests and reconstructions were performed by a single surgeon (K. H.). All patients underwent routine follow-up evaluations for two or more years postoperatively (median 44 months, range 25-76 months).

ORN was defined as nonhealing bone exposure of at least six months’ duration [8]. All patients suffered from diverse symptoms, including intractable pain and infection that required repeated antibiotic administration. Among them, 13 patients experienced eating difficulty due to severe trismus or a pathological fracture. Two patients required gastrostomy. For staging of ORN before surgery, the classification proposed by Lyons et al. [9] was applied in this study. In brief, stage 1: affected bone <2.5 cm; stage 2: asymptomatic affected bone >2.5 cm; stage 3: symptomatic affected bone >2.5 cm; and stage 4: affected bone with pathological fracture, orocutaneous fistula, or involvement of the inferior alveolar nerve.

The extent of mandibulectomy with adequate safety margins from the apparently osteolytic areas was determined by preoperative thin-slice computed tomography (CT). Although ethical approval was exempted because of the retrospective nature of this study, all subjects gave written informed consent to release clinical information.

The following epidemiological data were gathered retrospectively from the patients’ medical charts: age, sex, pathological diagnosis, primary tumor sites, types of RT, radiation dose, chemotherapy, surgery for the primary tumor, and perioperative antibiotics. Mandibulectomy defects were classified according to the “CAT classification” used in our previous reports [10,11]. Briefly, defects were classified on the basis of three anatomical landmarks: the mental tubercle [T], mandibular angle [A], and condyle [C]. The lesions that did not include tubercle, angle, and condyle were classified as mandibular “body”.

Early complications were defined as those that developed <30 days after ORN surgery. Late complications were defined as those that occurred one month or later after ORN surgery and included infections (local and distant), plate exposure, and recurrence (i.e., osteolysis at the resection margins). We enrolled patients in the present study who underwent routine follow-up panoramic radiography and CT during the two or more years after surgery to monitor for tumor recurrence or to confirm plate fixation and who did not have early tumor recurrence. Follow-up panoramic plain radiography or CT imaging was performed at least once every six months.

Statistical analyses were performed using R software (2011; R Development Core Team, Vienna, Austria). The groups were compared using Fisher’s exact test for categorical variables. A value of P<0.05 was considered to indicate statistical significance.

## Results

Clinical characteristics are shown in Table 1. Among the 15 patients, three (20%) had diabetes mellitus and six (40%) underwent resection of a primary tumor concomitant with free radial forearm flap reconstruction. All patients had stage 4 ORN before surgery. Acute inflammation due to infection immediately before surgery was not found in all patients. The nutrition status after surgery was good in all patients.

**Table 1:** Clinical characteristics of patients AC: adenocarcinoma; 5-FU: 5-fluorouracil; IMRT: intensity-modulated radiation therapy; NDP: nedaplatin; SCC: squamous cell carcinoma. * Extent of mandibulectomy was classified according to the CAT classification, described in detail in the text.

Case	Sex	Age	Pathological diagnosis	Primary site	Type of radiation therapy/dose (Gy)	Chemotherapy	Primary reconstruction	Neck dissection	Extent of mandibulectomy*
1	M	71	SCC	Oral	Conventional/61.5	-	Radial forearm	Bilateral	Body
2	M	58	SCC	Oropharynx	Conventional/70	Cisplatin	-	Unilateral	Body
3	M	70	SCC	Neck (unknown primary)	Conventional/66	Cisplatin	-	Unilateral	Body
4	M	62	SCC	Oropharynx	Conventional/70	Cisplatin/5-FU	-	-	A
5	F	80	SCC	Oral	IMRT/60	-	Radial forearm	Unilateral	AT
6	M	66	AC	Neck (unknown primary)	Conventional/60	-	-	Unilateral	A
7	M	65	SCC	Oropharynx	Conventional/60	Cisplatin/NDP	Radial forearm	Unilateral	A
8	M	64	SCC	Neck (unknown primary)	Conventional/81	Cisplatin/5-FU	-	-	A
9	M	63	SCC	Nasopharynx	Conventional/70	Cisplatin/5-FU	-	-	Body
10	M	63	SCC	Oropharynx	Conventional/70	Cisplatin	-	-	Body
11	M	74	SCC	Oropharynx	Conventional/66	Cisplatin/5-FU	Rectus abdominis	Bilateral	Body
12	M	57	SCC	Oropharynx	Conventional/66	Cisplatin	-	-	TT
13	M	78	SCC	Oropharynx	Conventional/60	-	Radial forearm	Unilateral	Body
14	M	77	SCC	Oral	Conventional/50	-	Radial forearm	Unilateral	A
15	M	57	SCC	Oropharynx	Conventional/70	Cisplatin	-	-	CA

The details of complications are shown in Table 2. Only one of the 15 patients had total flap necrosis. He underwent retransfer of a contralateral fibula flap, and his second postoperative course was good. Hence, the rate of total necrosis of the fibula flap was 6.3% (1/16 transferred fibula flaps). Other early complications comprised one case of arterial thrombosis one day postoperatively that was salvaged by reoperation and one case of partial necrosis (i.e., only cutaneous flap loss) for which bone flap engraftment produced a good result.

**Table 2:** Early and late complications ^a^Time interval between osteoradionecrosis surgery and the occurrence of late complications. ^b^Total dose per day. ^c^In this patient, the flap was sutured in the neck. ^d^In this patient, intraoral resuture was performed, and a bone flap was engrafted.

Patient	Early complication	Follow-up duration (months)	Late complication (months)^a^	Postoperative antibiotic duration (days)/dose (g)^b^
1	-	76	-	5/9
2	-	64	Distant infection (2)	3/9
3	-	50	Recurrence (3)	2/4.5
4	Total loss of fibula flap (retransferred fibula flap was engrafted)	49	-	7/9
5	-	46	-	5/9
6	-	46	-	5/6
7	-	45	Local infection (2)	5/6
8	Dehiscence of cutaneous flap sutured intraorally	44	Plate exposure (17)	5/6
9	Dehiscence of intraoral primary suture^c^	43	-	14/6
10	-	39	-	16/6
11	Arterial thrombosis (salvaged by reoperation)	39	-	15/6
12	-	38	Recurrence (12)/plate exposure (21)	20/6
13	-	32	-	9/6
14	-	28	-	8/6
15	Loss of cutaneous flap sutured intraorally^d^	25	-	8/6

Late complications were found in five of 15 patients (33.3%). The median value of the time intervals between ORN surgery and the occurrence of late complications was 7.5 months (range 2-21 months). The details were as follows: two infections (one local, one distant), two plate exposures, and two recurrences at the resection margins.

**Figure 1:**
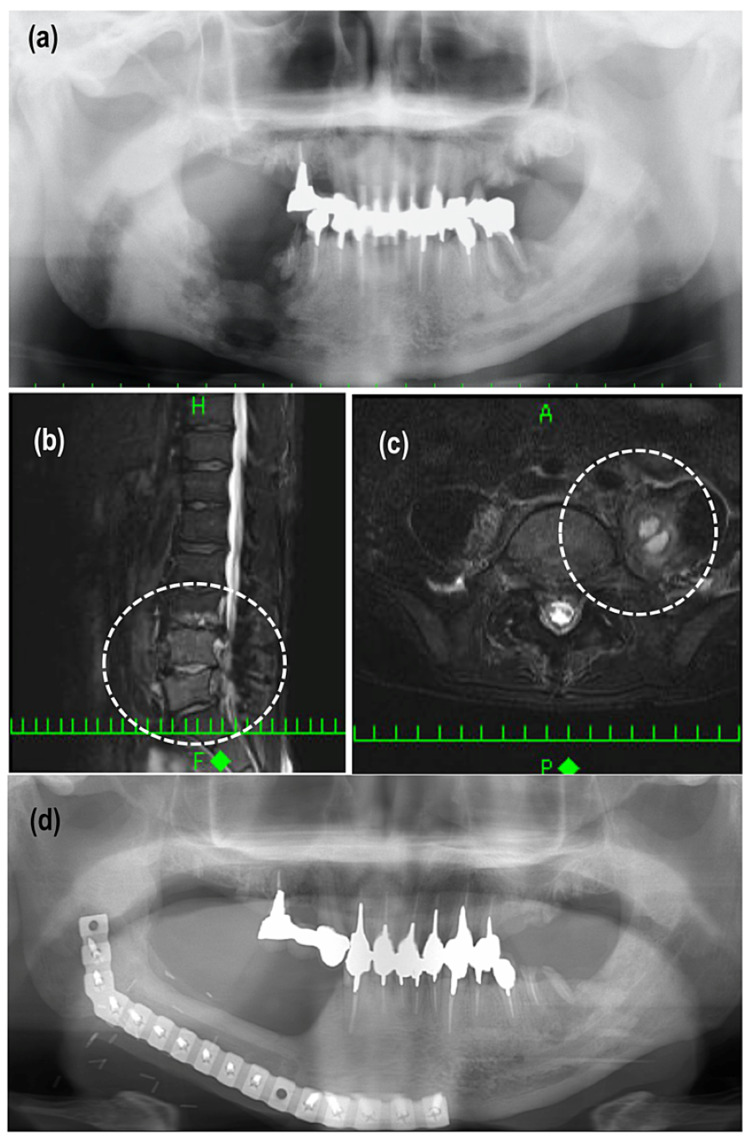
Distant infection in patient 2. (a) Preoperative panoramic radiographic image shows bone destruction close to the inferior border of the mandible. Distant infection occurred 82 days postoperatively. Magnetic resonance imaging revealed (b) pyogenic spondylitis and (c) an iliopsoas abscess. (d) Panoramic radiographic image 5 years after surgery shows excellent bone union.

The local infection which was evident by skin redness and drainage of pus occurred two months after surgery in patient 7 in the neck region. It was treated conservatively by antibiotic administration. The distant infection occurred in patient 3, who had diabetes mellitus and had undergone segmental mandibulectomy and fibula flap reconstruction for advanced ORN in the right mandible (Figure 1a). Sulbactam sodium-ampicillin sodium (3 g) was administered three times per day for three days postoperatively. Although his postoperative course was good, he experienced severe low back pain 82 days after the surgery. Magnetic resonance imaging revealed pyogenic spondylitis and an iliopsoas abscess (Figure 1b, 1c). Blood culture revealed *Streptococcus anginosus*. A postoperative distant infection was suspected, and antibiotic treatment was started. The distant infection healed, and the infection did not recur. Excellent bone union was confirmed on panoramic radiographic images five years postoperatively (Figure 1d).

**Figure 2:**
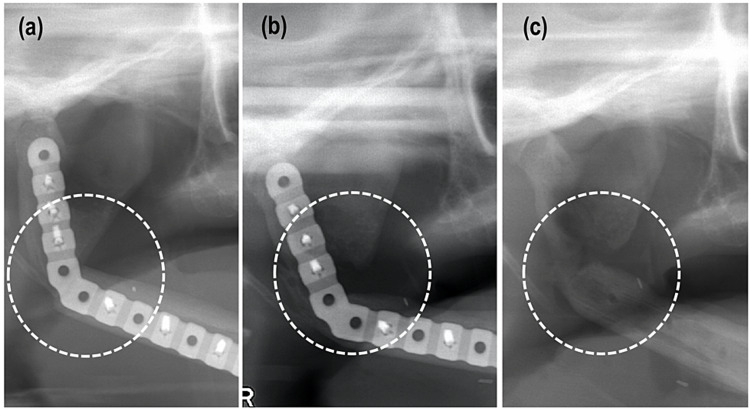
Recurrence at the resection margin in patient 12. (a) Panoramic radiographic images 1 month after surgery. (b) Bone resorption was evident 2 years after surgery. (c) After plate removal, bone union was confirmed.

Plate exposure occurred in two patients, at seventeen and 21 months after their surgery, respectively, Each underwent plate removal under general anesthesia, with good healing. Although recurrence was seen three and twelve months postoperative in patients 3 and 12, respectively, bone resorption stopped spontaneously, followed by adequate bone union fusion (Figure 2).

All patients received sulbactam/ampicillin after surgery. Administration duration was seven or fewer days in eight patients and eight or more days in seven patients. The two patients who experienced infection were treated with postoperative antibiotics for three and five days, respectively. There was no significant difference in the occurrence of late complications between the short (seven or fewer days) and long (eight or more days) postoperative antibiotic administration groups, although there were no infections in the long-administration group.

## Discussion

Although surgical intervention (e.g., radical debridement, well-vascularized tissue transfer) is essential for treating advanced ORN, the occurrence of complications is high because ORN surgery is difficult because of the vascular compromise and the presence of fibrosis and longstanding infection. In a recent report of a 10-year experience in a single center, the overall success rate of ORN reconstructive surgery was 92.3% [12], similar to that found in the current study (93.7%). Only a few studies have included 10 or more cases and evaluated long-term (two or more years postoperatively) outcomes of ORN reconstructive surgery (Table 3) [12-16].

**Table 3:** Literature review of late complications following osteoradionecrosis reconstructive surgery ^a^Not reported. ^b^Proportion in the fibula patients.

Study	Total number of primary flaps	Mean follow-up duration (months)	Early complications (%)	Late complication (%)
Bettoni et al. (2019) [9]	55 (Bone or composite flap 39/fibula 15)	48	34	20
Chang et al. (2001) [10]	30 (Bone or composite flap 24/fibula 17)	33	Flap loss 21	-^a^
Santamaria et al. (1998) [11]	Fibula 12	45	25 (Thrombosis 8, fistula 8, partial loss of skin paddle 8)	Recurrence 0
Sawhney & Ducic (2013) [12]	37 (Bone or composite flap 34/fibula 32)	54	24 (Loss of soft-tissue paddle 6, plate exposure 9^b^)	
Støre et al. (2002) [13]	17 (Bone or composite flap 16/fibula 7)	57	Fibula flap loss 28/minor complications 58	
Present study	Fibula 15	44	33 (Flap loss 6)	33

In the present study, the occurrence of late complications was 33.3% (5/15 patients). Although plate removal under general anesthesia was required in the two patients with plate exposure, healing after plate removal was good. Because thinning of the skin due to radiation fibrosis is often observed in ORN patients, plate exposure after surgery seems to be a relatively common late complication. Surgeons should explain to patients the possibility of plate removal after ORN reconstructive surgery.

Two cases of osteolysis from the resection margins were seen in the current study (13.3%, 2/15 patients). A previous study reported that postoperative residual or recurrent ORN is not a rare complication (25% occurrence rate) [17]. Determining the extent of the resection for ORN remains an unresolved issue [11]. One of the yet-to-be-answered questions is whether recurrence after ORN surgery is associated with insufficient resection of necrotic bone. Interestingly, Zaghi et al. [18] reported that the presence of residual necrotic bone at resection margins following segmental mandibulectomy did not correlate with the recurrence of ORN. All ORN recurrences have been found in viable resection margins, and there was no progression of ORN at the resection margins in residual nonviable bone [18]. Our previous histopathological study revealed that complete extirpation of necrotic bone is impossible in some cases, despite resection of the area of the destroyed bone with a wide safety margin [11]. Necrotic bone inevitably remains in severe cases with ischemia and acellularity caused by radiation damage. External stimuli, such as resection and screw insertion, may trigger osteolysis in the residual mandible that involves necrotic bone. A review that focused on osteolysis around total knee arthroplasty stated that necrosis and bacterial fragments might play a role in periprosthetic osteolysis [19]. Fortunately, the two cases of postoperative osteolysis in the current study stopped spontaneously or after plate removal. The underlying mechanism of, and risk factors for, osteolysis after ORN surgery need to be elucidated.

To the best of our knowledge, this is the first report to focus on distal infections after ORN reconstructive surgery. Our patient 3 developed pyogenic spondylitis and an iliopsoas abscess 82 days after ORN reconstructive surgery. The delayed infection after surgery has been previously reported. For example, the bacteremia after prosthetic joint replacement surgery occurred in 3.8% and the median duration between surgery and bacteremia was 1460 days [20]. In our case, blood culture revealed *Streptococcus anginosus* infection. Also known as the* Streptococcus milleri* group, the *S. anginosus* group includes *S. constellatus*, *S. intermedius*, and *S. anginosus*. These bacteria comprise the normal flora of the mouth, gastrointestinal tract, and genitourinary tract and are often associated with purulent infections [21]. There are reports about remote infection caused by *S. anginosus* group bacteremia [22,23], one of which occurred following dental cleaning [23]. In our case, there were several possible origins of a hematogenous infection with the *S. anginosus* group: (1) residual bacteria in the surgical field via the grafted fibula flap; (2) the presence of ORN in the left mandible; and (3) other origins (e.g., maxillary sinusitis, otitis media). In the present patients, there was no evidence of maxillary sinusitis, otitis media, or other infectious origins. Although bone resorption around the left first molar was evident (Figure 1a), there was no obvious infection of the left mandibular ORN area between before the surgery and the appearance of the postoperative bacteremia. The remote infection of residual bacteria in the surgical field via the grafted fibula flap seemed to be the most suspicious origin of the hematogenous infection with *S. anginosus*. However, the bacterial culture test of bone samples from primary surgery was not performed. So, we cannot affirm that the distant infection was caused by postoperative bacteremia from the surgical area.

As complete resection of necrotic tissues is difficult in severe ORN cases, ORN reconstructive surgery itself may pose a possible risk of transient bacteremia due to residual bacteria in the surgical field via transferred well-vascularized tissue. To prevent this hematogenous infection, perioperative antibiotic administration is likely critical. Although there seems to be no guideline about the types and duration of perioperative antibiotic administration for ORN surgery, we have referred to guidelines for diseases in other regions of the body. For example, the Infectious Diseases Society of America’s clinical practice guideline for the diagnosis and treatment of diabetic foot infections stated that the most appropriate duration of therapy is not well defined, but it is important to consider the presence and amount of residual dead and infected bone and the state of the soft tissues [24]. When a radical resection leaves no remaining infected tissue, only a short duration of antibiotic therapy (specifically, two to five days) is sufficient. In contrast, if infected or dead bone remains despite surgery, prolonged treatment is recommended (residual infection requires treatment for four to six weeks; residual dead bone requires treatment for more than three months) [24]. Although diabetic foot infections and ORN are not the same disease, the concept of changing the duration of perioperative antibiotic administration depending on the extent of residual necrotic bone after surgery should be applicable to ORN surgery. After experiencing patient 3, we changed the duration of perioperative antibiotic administration. Although there was no significant difference in the occurrence of all late complications between short- and long-duration perioperative antibiotic administration, no distant infections occurred after ORN reconstructive surgery in patients in the long-duration antibiotic group.

The current study, however, was retrospective and includes only a small sample. Hence, further investigation to elucidate the appropriate administration of perioperative antibiotics following ORN surgery is needed.

## Conclusions

Late complications after ORN reconstructive surgery are probably not uncommon since this surgery is difficult because of the vascular compromise and the presence of fibrosis and longstanding infection. Surgeons who perform ORN reconstruction should pay attention to the possibility of distant infections after ORN reconstructive surgery. Extirpation of infected tissues is essential for preventing delayed hematogenous infection following ORN reconstruction. Complete resection of necrotic tissue, however, is difficult in patients with severe radiation damage. In such cases, perioperative antibiotic administration should be adjusted based on the operative findings.
